# An Efficient Enzyme-Less Uric Acid Sensor Development Based on PbO-Doped NiO Nanocomposites

**DOI:** 10.3390/bios12060381

**Published:** 2022-05-31

**Authors:** Md Mahmud Alam, Abdullah M. Asiri, Mohammed M. Rahman

**Affiliations:** 1 Center of Excellence for Advanced Materials Research (CEAMR), King Abdulaziz University, P.O. Box 80203, Jeddah 21589, Saudi Arabia; mmalam1@kau.edu.sa (M.M.A.); aasiri2@kau.edu.sa (A.M.A.); 2 Chemistry Department, Faculty of Science, King Abdulaziz University, P.O. Box 80203, Jeddah 21589, Saudi Arabia

**Keywords:** PbO-doped NiO nanocomposites, uric acid sensor probe, sensitivity, real sample analyzing, health care safety

## Abstract

Here, the voltammetric electrochemical approach was applied to detect uric acid (UA) in a conductive sensing medium (phosphate buffer solution-PBS) by using PbO-doped NiO nanocomposites (NCs)-decorated glassy carbon electrode (GCE) performing as working electrode. The wet-chemically prepared PbO-doped NiO NCs were subjected to characterization by the implementation of XRD, FESEM, XPS, and EDS analysis. The modified GCE was used to detect uric acid (UA) in an enzyme-free conductive buffer (PBS) of pH = 7.0. As the outcomes of this study reveal, it exhibited good sensitivity of 0.2315 µAµM^−1^cm^−2^ and 0.2233 µAµM^−1^cm^−2^, corresponding to cyclic (CV) and differential pulse (DPV) voltammetric analysis of UA, respectively. Furthermore, the proposed UA sensor showed a wider detection (0.15~1.35 mM) range in both electrochemical analysis methods (CV & DPV). In addition, the investigated UA sensor displayed appreciable limit of detection (LOD) of 41.0 ± 2.05 µM by CV and 43.0 ± 2.14 µM by DPV. Good reproducibility performance, faster response time and long-time stability in detection of UA were perceived in both electrochemical analysis methods. Finally, successful analysis of the bio-samples was performed using the recovery method, and the results were found to be quite acceptable in terms of accuracy. Thus, the findings indicate a reliable approach for the development of 5th generation biosensors using metal-oxides as sensing substrate to fulfill the requirements of portable use for in situ detection.

## 1. Introduction

Generally, uric acid (UA) is produced through bio-metabolism of purine nucleotides and execrated from human blood serum via the renal pathway as urine. UA is a strong anti-oxidant and reducing agent in human blood serum [1]. Due to poor kidney dialysis function, UA levels in human blood can gradually increase, and can be responsible for perinatal risk of asphyxia, tumors, hyperuricemia, hyperthermia, pre-eclampsia, leukemia, cerebral ischemia, high blood pressure, atherosclerosis, Parkinson’s disease, kidney disease, high blood lipids and gout [2,3,4,5,6,7]. From the viewpoint of a healthy life, only the early diagnosis of uric acid in human blood can prevent millions of lives from being affected by dreadful diseases. Therefore, a reliable and convenient portable device for analysis of uric acid is necessary for the detection and diagnosis of the above-reported syndromes in humans. To date, distinct analytical approaches have been applied, including HPLC [8,9,10], electrochemical methods [11,12], fluorescence probes [13], and the enzyme method [14] for the reliable detection and quantification of uric acid. Among these uric acid detection methods, the electrochemical technique of voltammetry has more potential than others.

Recently, various Molecularly Imprinted Polymer (MIP) electron sensing substrates have been used in the development of UA sensors, applying distinct electrochemical detection methods. These materials include graphene-doped chitosan [15], MIPCPE [16], UA-imprinted-poly(hydroxyethyl-methacrylate-methacryloyl-l-cysteine-methylester)-Fe^3+^[poly(HEMA-MAC)-Fe^3+^] NPs [17], uric-acid-imprinted polypyrrole [18], carbon-enwrapped nickel NPs [19], MIP-nano-porous Au-leaf [20], polypyrrole-based MIP [21] and conductive-azopolymer based MIP [22]. Photoluminescence spectroscopy [23], surface plasmon resonance [24] and photo-electrochemical technique [25] have also been employed to develop UA sensors. From all these methods, cyclic voltammetry (CV) has been found to demonstrate the most potential and greatest reliability. Therefore, this type of potential dynamic electrochemical measurement has been applied to detect UA based on various modified electrodes, and exhibited wider LRD, such as 0–50.0 µM for Nafion^®^-coated carbon-paste [26], 2.5 µM–0.03 mM for pyrolytic graphite (PGE) [27], 20.0 µM–1.0 mM for solar graphene [28], 1.0 µM–0.3 mM for carbon paste (CPE) decorated with iron(II) phthalo-cyanine [29], 2.0 µM–0.6 mM for PEDOT-modified gold [30], and 6 µM–0.1 mM for methylene-blue as adsorbed on phosphorylated zirconia–silica composite [31]. These dynamic ranges for uric acid detection are suitable for the analysis of uric acid in human serum (0.24~0.51 mM in the adult male and 0.16~0.43 mM in the adult female). The interference effects of those sensors have been investigated precisely. The reproducibility, stability and response times of these reported UA sensors have not been evaluated. Except for these parameters, the inspected sensors are adequately fit for application for the purpose of developing a tiny and portable uric acid sensor probe. Thus, the aim of this study is to advance the development of a UA sensor based on nanocomposites of metal oxides capable of analyzing levels of UA in human serum. The assembled sensor should fulfil all requirements and parameters to establish reliability.

PbO is an n-type semiconductor, with tetragonal and orthorhombic phases of optical band-gap 1.9–2.2 eV and 2.6 eV, respectively. Therefore, PbO has been reported as a voltammetric electrochemical sensor to detect glucose [32]. NiO also belongs to the p-type semiconductor group, with an optical band-gap of 3.2 eV and is especially efficient in numerous sensing applications including M-tolyl hydrazine [33], L-glutamic acid [34], choline [35], and kanamycin [36], via an electrochemical approach. It has been stated that PbO and NiO have potential electrochemical properties suitable for sensing applications.

Doped nanocomposite materials have gained a great deal of attention due to their chemical, structural, physical, and optical properties. They can demonstrate large active surface area, high stability, high porosity, and permeability, directly dependent on the structural morphology prepared by reactant precursors for these materials in basic medium at low-temperature. Here, NCs were synthesized by a wet-chemical method using reducing agents in ambient conditions. This technique and the method have several advantages, including simple preparation, accurate control of the reactant temperature, easy handling, one-step reactions, high surface area, and high porosity. Optical, morphological, electrical and chemical properties of PbO-doped NiO nanocomposites are of huge significance compared to undoped PbO or NiO materials. Non-stoichiometry, characterized mostly by oxygen vacancies, provides the conducting nature in the PbO-doped NiO nanocomposites. The formation energy of oxygen vacancies and metal interstitials in semiconductors is very low and thus these defects form easily, resulting in the experimentally elevated conductivity of NCs compared to undoped materials. PbO-doped NiO nanocomposites have also attracted considerable interest due to their potential applications in fabricating opto-electronics, electro-analytics, selective detection of assays, sensor devices, hybrid-composites, electron-field emission sources for emission exhibits, biochemical detection, and surface-enhanced Raman properties, etc. PbO-doped NiO nanocomposites offer improved performance due to their large active surface area which increases the conductivity and current responses of PbO-doped NiO NCs/Nafion/GCE assembly during electrochemical investigation.

Therefore, this study was conducted into the development of a voltammetric electrochemical sensor for reliable detection of UA in buffer solution. It was seen to perform appreciably and outstandingly. In the near future, this voltammetric electrochemical approach for diagnosis of numerous bio-chemicals in human fluid (urine and blood serum) will be useful for identifying dangerous physiological syndromes in humans.

## 2. Experimental

### 2.1. Materials and Methods

In this study, Sigma-Aldrich (Steinheim am Albuch, Germany) supplied the precursors of lead (II) chloride PbCl_2_ (CAS 7758-95-4) and Nickel (II) chloride hexahydrate NiCl_2_·6H_2_O (CAS 7791-20-0), which were used to prepare PbO-doped NiO nanomaterials. Auto-clave assisted co-precipitation was applied in 10.5 pH medium. Ammonium hydroxide (CAS 1336-21-6), uric acid (UA; CAS 69-93-2), mono-sodium phosphate (7558-80-7), di-sodium phosphate (CAS 7782-85-6), and adhesive glue (5% Nafion; CAS 31175-20-9) were obtained at analytical grade from the supplier (Merck—Darmstadt, Germany) to conduct this analysis. The XPS (Thermo—Dreieich, Scientific Germany, A1-k-α1 radiation sources and 300.0 µm beam size investigated at 200.0 eV) was applied at 10-8 Torr pressure on the prepared micro-structures (nano-materials) to analyze the ionization states and the binding energy of existing atoms. Later, the structural and elemental compositions of prepared metal oxides were confirmed by using FESEM and EDS analysis; instrument model JEOL, JSM-7600F (Tokyo, Japan) was used for this purpose. The crystallinity and particle size of the prepared PbO-doped NiO nanomaterials were evaluated using a powder X-ray diffractometer. Finally, the potentiostats/galvanostats (Metrohm Autolab Modules- PGSTAT128N, Herisau, Switzerland) was used as the constant potential supply source for the sensor application.

### 2.2. Wet-Chemical Preparation of PbO-Doped NiO NCs

For the preparation of doped nanostructure materials, the wet-chemical (co-precipitation) method was used. The advantage of this process is to prepare the proper shape and size of doped or un-doped materials at nano-level scale. Here, the PbO-doped NiO nanomaterials were prepared using a co-precipitation technique with the analytical grade PbCl_2_ and the NiCl_2_·6H_2_O salt. The initial preparation of this material used deionized water to form 0.1 M solution of PbCl_2_ and NiCl_2_·6H_2_O separately in two volumetric flasks of 100.0 mL. Then, a 200.0 mL beaker was used to mix these solutions of 50.0 mL each and stirred rigorously to ensure proper mixing. For the co-precipitation process of these metal ions into the solution, the 0.1 M NH_4_OH was drop-wise mixed to increase the pH to above 10.5. As we know, over pH 10.5, the dissolved metallic ions would be co-precipitated as metal hydroxides to form the doped nano-crystals of aggregated Pb(OH)_2_·Ni(OH)_2_. Later, the final mixture in the beaker was kept inside an auto-clave reactor, where the temperature was fixed at 80 °C for 6 h. The resultant mixture in the beaker was kept for 6 h under this condition to complete the co-precipitation in the synthesis of doped nanostructure materials. Then the precipitate was decanted and separated using filter paper. The precipitate was washed with ethanol, acetone, and water to remove all organic and inorganic parts as well as unreacted components from the prepared sample. The probable chemical reactions involved in the co-precipitation process are presented as follows: (Equations (1)–(4)),

NH_4_OH_(s)_ → NH_4_^+^_(aq)_ + OH^−^_(aq)_ (1)

PbCl_2(s)_ → Pb^2+^_(aq)_ + 2Cl^−^_(aq)_ (2)

NiCl_2(s)_ → Ni^2+^_(aq)_ + 2Cl^−^_(aq)_ (3)

Pb^2+^ + Ni^2+^_(aq)_+4OH^−^_(aq)_+nH_2_O → Pb(OH)_2_·Ni(OH)_2_·nH_2_O ↓ (4)

At this stage, the prepared aggregated nano-crystals of Pb(OH)_2_·Ni(OH)_2_·nH_2_O were dried in an oven at 120 °C for 12 h. The nanocrystals were finally calcined using the muffle furnace at 500 °C. The prepared metal hydroxides were oxidized to metal oxides in the presence of oxygen flow. It is proposed that the following reaction [Reaction (5)] formed the metal-oxide-doped nanostructure material during drying in the muffle furnace. The calcined doped nanostructure materials were then characterized for crystallinity, morphology, size, elemental analysis, band-gap energy, etc., using various conventional methods.

Pb(OH)_2_ + Ni(OH)_2_ → PbO·NiO+2H_2_O (5)

### 2.3. GCE Modification by PbO-Doped NiO NCs

For the fabrication of GCE, the key objective of this study was to modify the electrode surface with prepared PbO-doped NiO NCs for application in the development of an efficient working electrode for the proposed uric acid electrochemical sensor probe. The tiny GCE surface area (0.0316 cm^2^) was modified with a slurry of PbO-doped NiO NCs, prepared in the ethanol solution. Then the modified electrode was kept in an ambient condition during air drying for 10 min. For improved stability of the deposited layer on the GCE, a conducting coating binder of Nafion was used suspended as 5% in ethanol. Thus, the net-type binder was used as a chemical glue to stick the nanomaterials on the GCE surface for electrochemical analysis. This electrode fabricated with NCs and Nafion was air dried for 30 min to stablize the thin surface of the doped nanomaterials on the electrode. Finally, the electrode was prepared with PbO-doped NiO NCs as the PbO-doped NiO NCs/Nafion/GCE sensor probe for practical use in electrochemical investigation. With this working electrode, the Pt-wire and Ag/AgCl(sat. KCl) were used as counter and reference electrodes in the analysis. Then the analyte solution (UA) was used to prepare a series of solutions ranging from 0.15~1.35 mM and applied to the electrochemical characterization of the assembled sensor. Mono- and disodium phosphate solution (0.1M) were diluted into deionized water for the formation of the phosphate buffer solutions with different pH values ranging from 5.7 to 8.0. Finally, 30.0 mL samples of these prepared phosphate buffer solutions were used to prepare UA solution with various concentrations in the range of 0.15–1.35 mM, to carry out the electrochemical investigation.

## 3. Results and Discussion

### 3.1. Characterization of PbO-Doped NiO NCs by FESEM and EDS

**Figure 1:**
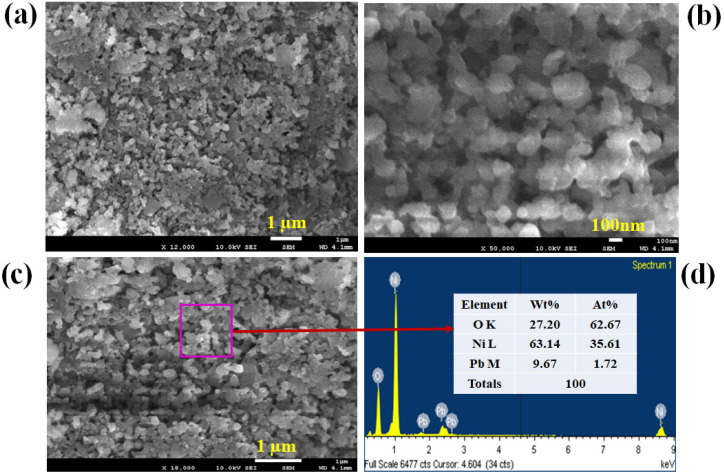
FESEM and EDS investigation of PbO/NiO nanomaterials. (**a**,**b**) Low and high magnification images of prepared PbO/NiO nanomaterials obtained from FESEM, (**c**) the magnified image of PbO/NiO nanomaterials from EDS analysis, and (**d**) atomic composition PbO/NiO NCs.

**Figure 2:**
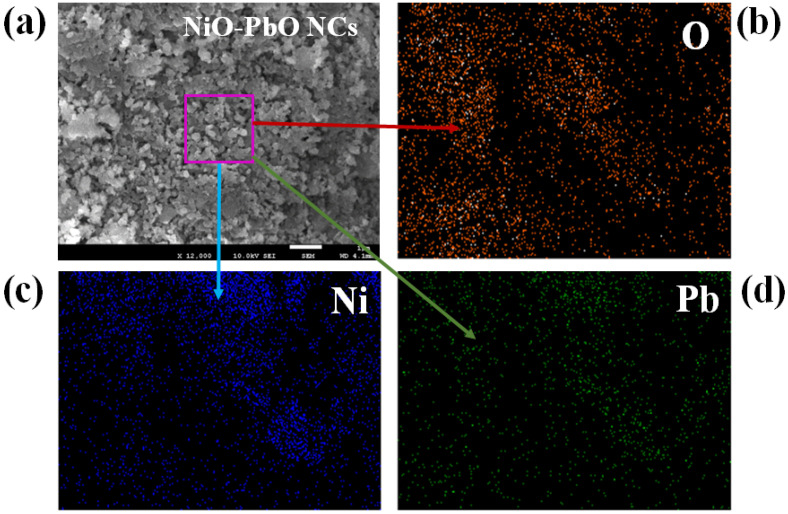
EDS mapping of PbO-doped NiO nanocomposites. (**a**) SEM image of PbO-doped NiO NCs, (**b**) Oxygen mapping, (**c**) Nickel mapping, and (**d**) Lead mapping.

The PbO/NiO nanomaterials were prepared in pH 10.5 alkaline medium applying a wet-chemical (co-precipitation) method. To enable investigation of PbO/NiO nanomaterials in terms of morphology as well as composition, samples were subjected to FESEM and EDS analysis, as shown in Figure 1. As observed from the low-to-high magnification FESEM images presented in Figure 1a,b, the nanocrystals of PbO/NiO nanomaterials are aggregated in such a way that it resembles a flower-type, but not exactly flower shape. Therefore, the synthesized nanomaterials are named nanocomposites (NCs). A comparable image was obtained from the EDS analysis report, illustrated in Figure 1c. The analysis of elemental compositions of PbO/NiO nanocomposites (NCs) by EDS investigation revealed O = 27.2%, Ni = 63.14% and Pb = 9.67% by weight. Based on the elemental compositions, it can be asserted that the synthesized nanomaterials are PbO-doped NiO NCs. Thus, this characterization confirmed the existence of Pb, Ni, and O in the prepared NCs sample. The EDS-mapping of each elements and presented in the Figure 2; mapping analysis reveals the presence of O, Ni, Pb elements in the selected SEM image of PbO-doped NiO NCs.

### 3.2. XRD Analysis of PbO-Doped NiO NCs

**Figure 3:**
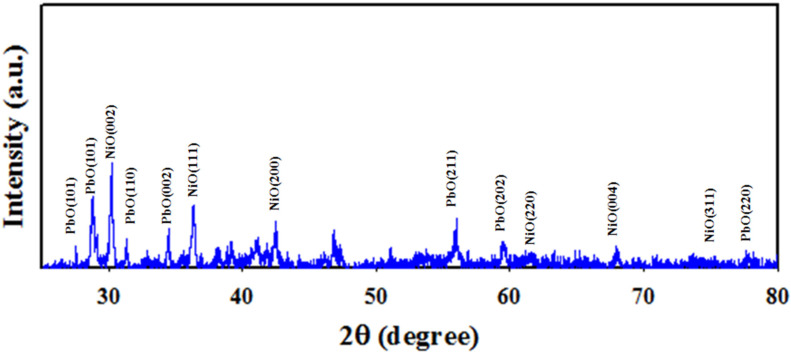
XRD analysis of PbO-doped NiO NCs performed at 2θ = 20–80°.

The XRD analysis of PbO-doped NiO NCs was performed at a range of 2θ = 20~80° represented in Figure 3. Prepared NCs consisted of crystalline phases of PbO and NiO only, as seen in Figure 3. As obtained, the plans of (100), (101), (110), (002), (211), (202), and (220), of PbO were observed and identified by JCPDS no. 0005-0561 and previous authors [37,38]. The crystalline plans of NiO including (002), (111), (200), (220), (004), and (311) are also confirmed in Figure 3, compared with JCPDS no. 0047-1049 and the reported article on NiO [39]. The average nano-grain size of CNs was calculated applying Debye-Scherer’s formula presented in (6), and calculated size is 21.50 nm obtained from the Ni(002).

D = 0.9 λ/(βcosθ)(6)

Here, λ = wavelength at 1.5418 Å of X-ray radiation, β = width Ni (002) peak at half (FWHM) position. Thus, the XRD analysis identified crystalline plans of PbO and NiO that completely agreed with FESEM and EDS analysis.

### 3.3. XPS Analysis of PbO-Doped NiO NCs

**Figure 4:**
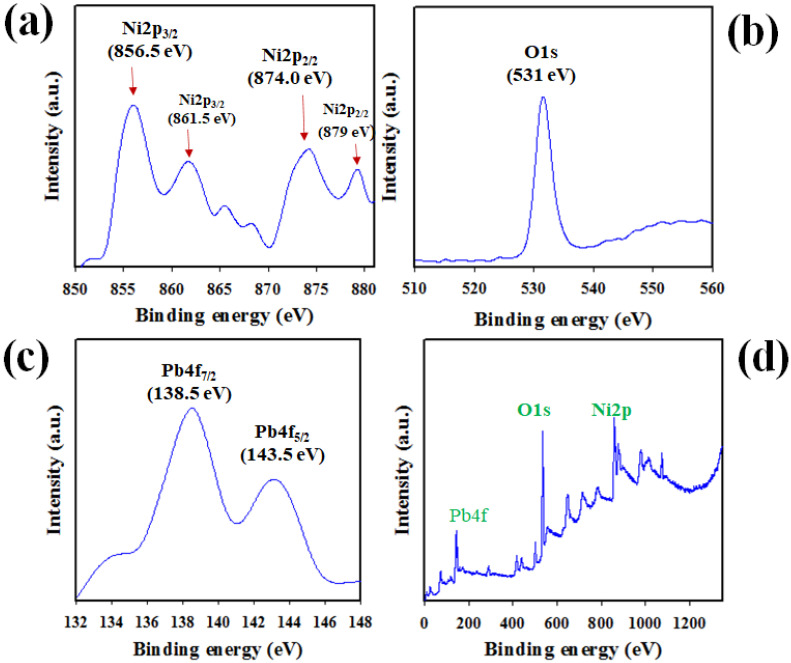
(**a**) The spin orbitals of Ni2p level, (**b**) the orbital O1s, (**c**) the spin orbitals of Pb4f level, and (**d**) XPS spectrum of PbO-doped NiO NCs.

Figure 4 represents the XPS orbitals of synthesized PbO-doped NiO NCs, and consists of O1s, Ni2p and Pb4f orbitals. Peaks associated with the presence of impurities were not identified, as illustrated in Figure 4. As shown in Figure 4a, the Ni2p orbital split to form Ni2p_3/2_ and Ni2p_1/2_ spin orbitals, positioned at 856.5 and 874.0 eV with spin separation of 17.5 eV. Thus, the resultant values can be characterized for presence of Ni^2+^ oxidation in the prepared PbO-doped NiO NCs. Additionally, two satellite spin orbitals of Ni2p-level can be perceived in Figure 4a located at 861.5 and 879.0 eV, corresponding to Ni2p_3/2_ and Ni2p_1/2_ spin-orbitals, respectively, with binding energy separation of 17.5 eV. Therefore, the main and satellite peaks both confirmed 2+ oxidation of Ni in the prepared samples [40,41]. The O1s XPS spectrum as in Figure 4b presents an peak at 531.0 eV, indicating O^2−^ ion associated with the Ni-O and Pb-O bonds in the PbO-doped NiO NCs [42]. Also, the Pb4f orbital shows two spin orbitals of Pb4f_7/2_ and Pb4f_5/2_ located at 138.5 and 143.5 respectively and separated by 5 eV spin energy, which is recognized for oxidation of Pb^2+^ in PbO-doped NiO NCs, as seen in the Figure 4c [43,44]. Overall, the XPS analysis reflected the analogous information obtained from the FESEM, EDS and XRD investigation of prepared NCs.

### 3.4. TEM Analysis of PbO-Doped NiO NCs

**Figure 5:**
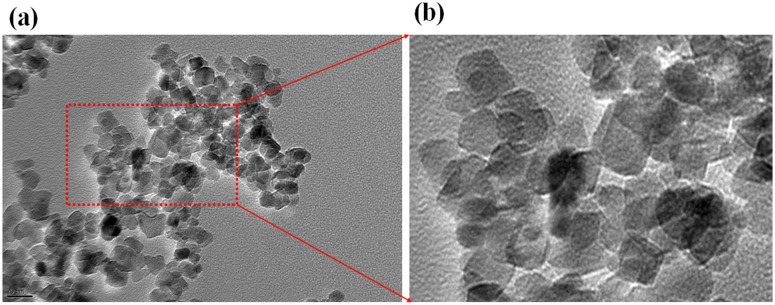
TEM analysis of PbO-doped NiO nanocomposite. (**a**) low-magnified image and (**b**) high-magnified image.

In this approach, the morphology of the PbO-doped NiO nanocomposite material was characterized by recording TEM images, and is presented in Figure 5a,b. It can be seen that PbO-doped NiO NCs are present as particle shapes of diameter in the range 15–28 nm. The average diameter of doped material was 17.5 nm. This observation implies that PbO-doped NiO NCs would provide a large surface area due to the small size (nano-scale), potentially helpful for the efficient sensing of uric acid by electrochemical methods.

### 3.5. Characterization of Working Electrode (WE)

**Figure 6:**
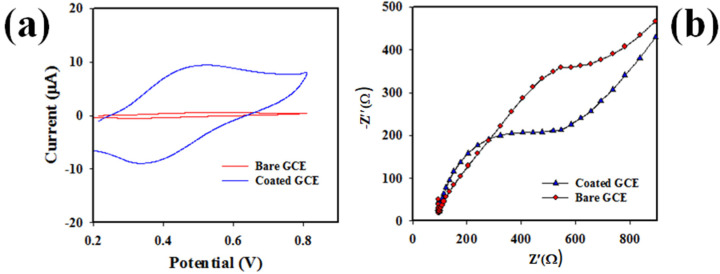
(**a**) The electrochemical characterization of PbO-doped NiO NCs WE and bare GCE at oxidation and reduction of 0.6 mM UA in pH = 7.0 buffer. (**b**) the Nyquist plot of UA to measure impedance of coated and bare GCE in 0.6 mM UA, pH = 7.0 buffer.

The GCE coated with PbO-doped NiO NCs was characterized by applying a voltammetric electrochemical approach to oxidize and reduce 0.6 mM UA in pH = 7.0 PBS (phosphate buffer solution), shown in Figure 6a. As established in Figure 6a, the oxidation and reduction peak point current of 0.6 mM UA obtained its maximum for PbO-doped NiO-coated GCE compared to bare GCE. Oxidation to reduction peak separation for bare GCE (∆Ep = infinity) was higher compared to coated GCE (∆Ep = +0.12 V). Therefore, the narrow oxidation–reduction peak separation potential (∆Ep) of coated GCE confirmed the higher electron mobility of modified GCE, as similar observations have previously indicated [45]. Impedance spectroscopy is can measure the electron transfer rate of an electrode. For this purpose, the modified GCE based on PbO-doped NiO NCs was subjected to impedance testing using 0.6 mM UA in 7.0 pH buffer solution, as recorded in Figure 6b. As shown in the Nyquist plot (Figure 6b), the semi-circle diameter is small for the coated GCE compared to bare GCE.

Therefore, the coated GCE had the lesser charge transfer resistance (R_tc_) in the oxidation of UA. In other word, the WE (working electrode) based on PbO-doped NiO NCs/GCE demonstrated high electron mobility capacity, a similar concept has been reported by previous authors [46,47].

**Figure 7:**
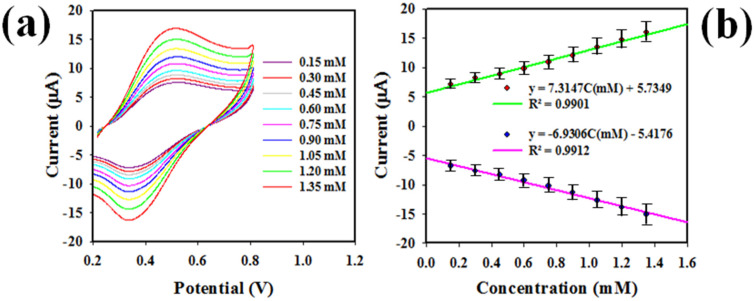
(**a**) The SR of modified GCE in electrochemically analysis of 0.6 mM UA, in pH = 7.0 buffer solution, (**b**) the square -root of SR versus oxidation-reduction peak point currents of UA.

Figure 7a presents the scan-rate (SR) of GCE modified by PbO-doped NiO NCs using 0.6 mM UA applying the voltammetric electrochemical approach in pH = 7.0 buffer solution. As demonstrated in Figure 7a, the peak pint current for oxidation (+0.5 V) and reduction (+0.38 V) are linearly shifted at the 25~300 mV/s range. In the Figure 7b, the square roots of SR are plotted against the peak point’s oxidation and reduction current, and revealed to be linear; the equations of resulting lines are i_p_ = 42.673 (SR)^0.5^ − 4.9664 (R^2^ = 0.9914) for oxidation and i_p_ = 37.904 (SR)^0.5^ + 4.0735 (R^2^ = 9937). Thus, the good linearity of oxidation and reduction current suggest that the electrochemical oxidation and reduction of UA by coated GCE at 0.6 mM concentration of UA in pH = 7.0 buffer is a controlled diffusion process.

### 3.6. Electrochemical Detection of UA by Voltammetry Approach

**Figure 8:**
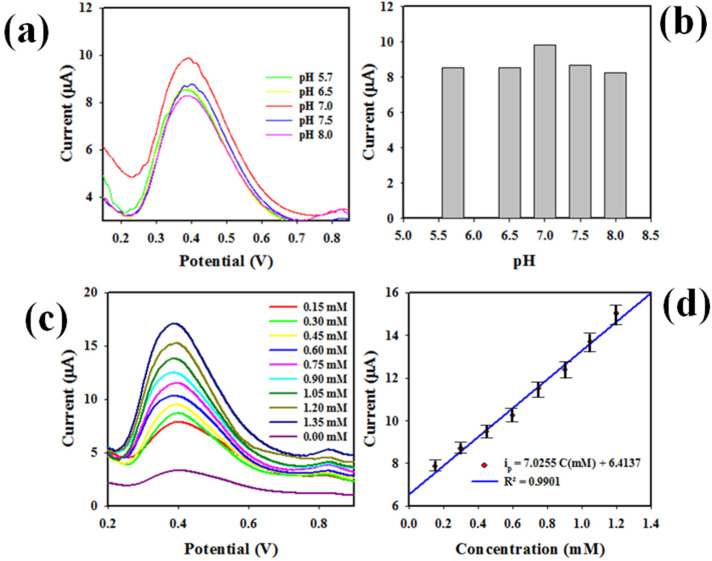
Detection of target chemicals with PbO-doped NiO NCs. (**a**) pH optimization DPV analysis of UA using PbO-doped NiO NCs/GCE electrode, (**b**) bar diagram of pH optimization, (**c**) DPV of UA based on modified GCE in buffer solution of pH 7.0, (**d**) calibration of UA sensor executed from peak current versus concentration of UA using the data from Figure 6c.

The voltammetry electrochemical detection method of a biochemical, such as differential-pulse voltammetry (DPV), is well-known and reliably applied technique. In this study, the DPV method was applied to analysis of 0.6 mM UA solution in the buffer medium of distinct pH (5.7~8.0) value based on PbO-doped NiO NCs coated on GCE; the data are presented in Figure 8a.

As perceived, UA showed the highest peak current at pH 7.0 in the oxidation, as the bar diagrams show in Figure 8b. Therefore, pH 7.0 can be considered the optimum pH value to detect UA in the DPV method. Next, the UA at 0.15 to 1.35 concentration range was subjected to DPV analysis using the WE based on PbO-doped NiO NCs/GCE, and the resulted outcomes are explored in Figure 8c. The peak point currents in the oxidation of UA increased with corresponding higher concentrations of UA in pH = 7.0 buffer solution. Thus, a calibration for UA detection can be established by plotting concentration versus current, demonstrated in the Figure 8d, which is found to be linear (Regression co-efferent; R^2^ = 0.9901) and expressed with the equation of i_p_(µA) = 7.0255C(mM) + 6.4137. The concentration ranges 0.15~1.35 mM, where concentration versus current was linear, are defined as the detection range (LDR) for UA in buffer. Obviously, the resultant LDR is broad and well-fitted to analysis of normal UA concentration ranges in human serum and urine. The slope of calibration of the UA sensor as seen in Figure 8d was used to calculate the sensor sensitivity, equal to 0.2233 µAµM^−1^cm^−2^, a value confirming the high sensitivity of the proposed UA sensor. A signal/noise = 3 was applied to calculate the limit of detection (LOD) of UA, an appreciable LOD of 43 ± 2.14 µM.

**Figure 9:**
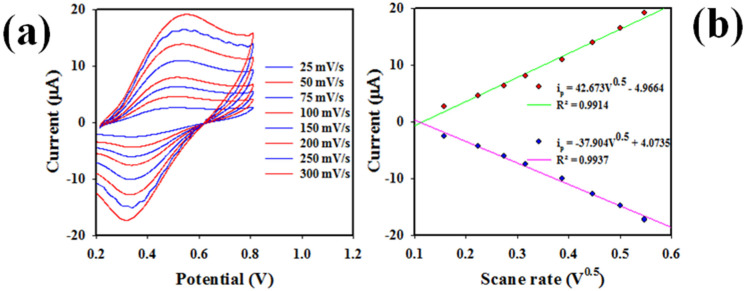
(**a**) Cyclic voltammetric analysis of UA at a range of concentrations (0.15–1.35 mM) in 7.0 pH buffer solution in presence of 0.6 mM K_3_Fe (CN)_6_ (0.1M PBS), and (**b**) calibration of UA sensor based on the analysis of CV, where peak oxidation and reduction currents are collected from Figure 7a.

Another promising method, CV was applied to analyze UA at a range of concentrations (0.15~1.35 mM) in buffer solution of pH = 7.0, as shown in Figure 9a. Analogous information as in Figure 8c can be perceived (the peak oxidation and reduction currents increased and decreased with increasing UA concentration in the testing buffer phase). Calibration plots were identified using peak currents versus concentration, as revealed in Figure 9b, which can be expressed by linear-equations i_p_(µA) = 7.3147 C(mM) + 5.7349 (R^2^ = 0.9901) for the oxidation of UA and i_p_(µA) = 6.9306 C(mM) − 5.4176 (R^2^ = 0.9912) for the reduction of UA, respectively, at a concentration ranges of 0.15~1.35 mM. Thus, the 0.15~1.35 mM UA concentration range is also considered to be the detection range (LDR) for UA in the cyclic voltammetry electrochemical approach, as similar to that obtained in Figure 8d for DPV analysis of UA. Considering the LDR slope as shown in Figure 9b, and the active surface area of GCE (0.0316 cm^2^), sensitivity for CV was calculated to a value of 0.2315 µAµM^−1^cm^−2^, very close to the DPV method (0.2233 µAµM^−1^cm^−2^). The limit of detection (41 ± 2.05 µM) was estimated applying signal/noise = 3, and closely matched the DPV method. Therefore, it can be predicted that the developed UA sensor based on PbO-doped NiO NCs/GCE is reliable and efficient, since both methods (DPV and CV) show substantial and analogous performances in UA detection in buffer phase at 0.6 mM UA concentration.

**Figure 10:**
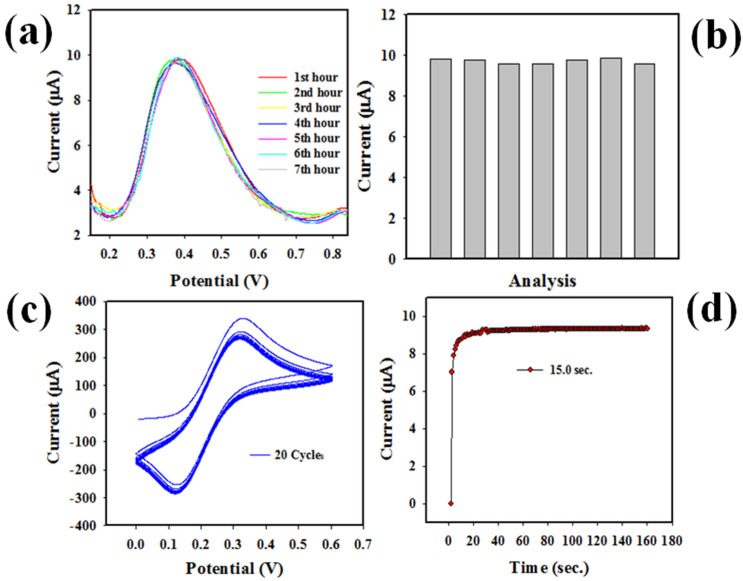
Optimization of sensor. (**a**) UA sensor reproducibility test, (**b**) reproducibility bar diagram of UA sensor, (**c**) performing stability test, and (**d**) response time of UA sensor.

To establish the reliability of the DPV sensor in analyzing UA, repeatability was tested at 0.60 mM UA in the pH = 7.0 buffer solution, presented in Figure 10a,b (bar diagram). As perceived, the data obtained from DPV analysis of UA are completely indistinguishable from the experimental conditions. Thus, it is confirmed from Figure 10a,b that the assembled UA sensor can analyzeUA reliably.

The sensor stability, which is another sensor reliability measuring parameter, was tested at 0.1 mM Fe(CN)_6_^3−/4−^ in a pH = 7.0 buffer solution, shown in Figure 10c. CV analysis showed that the proposed UA sensor demonstrated good stability at 20 cycles. Therefore, this test confirms the long-term performance ability as well as the reliability of the voltammetric electrochemical analysis of UA based on PbO-doped NiO NCs/GCE in buffer solution. The response time, another sensor efficiency measuring criterion, is defined as the period needed to complete an electrochemical analysis of UA by the proposed UA sensor. Thus, necessary parameter was tested using 0.6 mL UA in the pH = 7.0 buffer by applying an electrochemical approach (three-electrode based system), demonstrated and presented in Figure 10d. Fast response times around 15 s were found, which indicated the high efficiency as well as the faster response of this UA sensor with the fabricated electrode.

**Figure 11:**
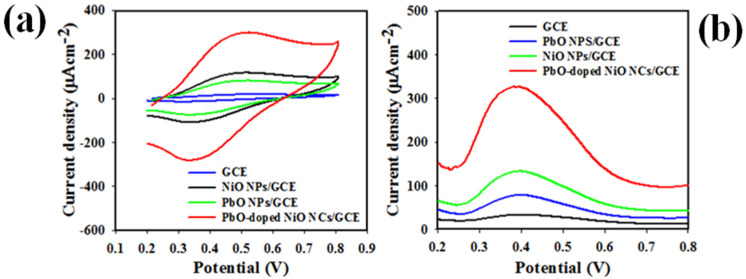
The control experiments for UA applying CV and DPV. (**a**) Current density versus applied potential of UA responses at 0.6 mM concentration for constituting single metal oxides compared with binary NCs of PbO-doped NiO applying CV and (**b**) similar experiments for UA applying DPV.

To establish the logic for using binary NCs of PbO-doped NiO as electron sensing substrate for UA, rather than the single constituting metal-oxides PbO and NiO, the control experiments were performed as presented in Figure 9. As shown in Figure 11a, GCEs without coating and those coated with NiO, PbO and PbO-doped NiO NCs were subjected to analysis using UA at 0.6 mM concentration in buffer medium of pH 7.0; PbO-doped NiO NCs on GCE exhibited the highest oxidation and reduction peak current points. The highest electrochemical activity of PbO-doped NiO NCs is helped the doping of NiO by PbO, which enhanced the reactive surface area of prepared NCs, evidently shown in the EDS analysis (Figure 1d). On the other hand, the prepared NCs of PbO-doped NiO showed high crystallinity with nano-level grain size as illustrated in Figure 3, which might be another cause of higher reactive surface area in the prepared NCs. Thus, in both plots (Figure 11a,b), UA showed high electrochemical responses in terms of current density versus potential.

**Figure 12:**
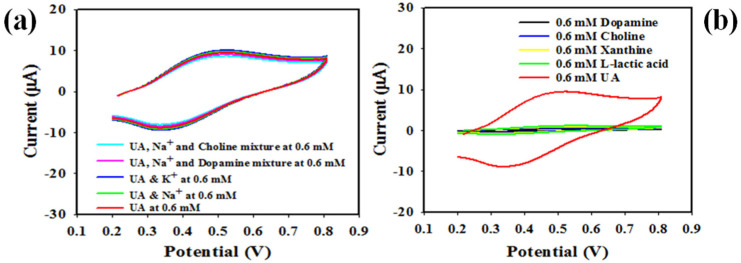
(**a**) Interference effect of UA sensor tested at 0.6 mM concentration of each component in a buffer medium of pH 7.0. (**b**) Selectivity study of UA sensor probe in identical condition in presence of various chemicals.

To test the reliability of the UA sensor based on PbO-doped NiO NCs coated on GCE, the interference effect of the assembled sensor was tested, as shown in Figure 12a. As illustrated in the plot, UA was analysed applying the CV electrochemical technique in the presence of Na^+^, K^+^, dopamine, and choline, and it was clearly observed that in the presence of these interfering chemicals, the electrochemical response of UA was not affected. Therefore, it can be concluded that the assembled UA sensor suffers no interference effect and is selective to UA only. For these experiments, 0.6 mM of each component was used in a butter medium of pH 7.0. The sensor probe with PbO-doped NiO NCs/GCE was also tested for its selectivity in the presence of other chemicals including dopamine, choline, xanthine, lactic acid, in identical conditions (0.6mM), presented in Figure 12b. It can be observed that the sensor probe PbO-doped NiO NCs/GCE exhibited the highest electrochemical process response in the presence of UA in identical conditions compared to other chemicals.

**Scheme 1:**
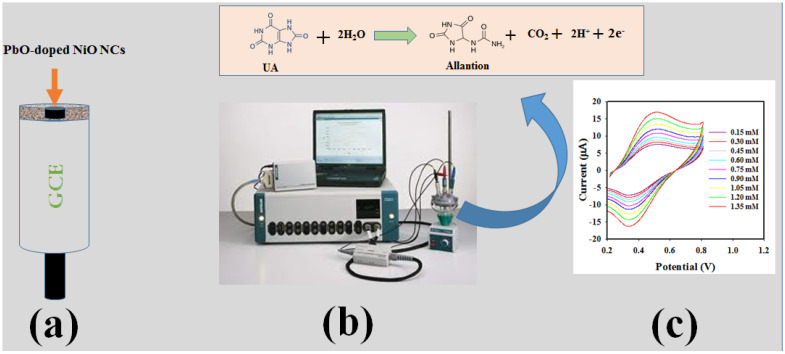
Schematic presentation of UA electrochemical sensor applying voltammetric electrochemical approach. (**a**) GCE modification with PbO-doped NiO NCs, (**b**) Instrumental arrangement of sensor Auto-lab, and (**c**) the resulting data from the analysis.

Scheme 1a is represents the sensor fabrication process using GCE and PbO-doped NiO NCs. As illustrated in Scheme 1b, UA molecules were absorbed on the GCE surface modified by PbO-doped NiO NCs and oxidized to Allantion. The oxidation of UA generated protons and electrons as illustrated. The peak currents obtained by cyclic voltammetric analysis of UA increased with increasing 0.6 mM UA concentration, indicated in Scheme 1c; analogous information has been published in the previous reports [48,49].

A comparison as in Table 1 [12,13,14,26,27,30,31,48,49,50,51,52,53,54] is illustrated to clarify the scientific evidence of this study based on LDR, LOD and sensitivity of the sensor. It is clearly demonstrated that the studied UA sensor performed better, and it is clearly observed that our investigated UA sensor PbO-doped NiO NCs–GCE performed well enough to analyze human serum (0.24~0.51 mM in the adult male and 0.16~0.43 mM in the adult female) perfectly. On the other hand, the most of the articles mentioned in Table 1 is failed to include the analysis of human serum in real sample validation. Among those, some may be fit for human use but have not been investigated properly in terms of sensor parameters such as sensitivity and LRD. Additionally, other sensor parameters including response time, stability, reproducibility, and sample meta-analysis have been seldom addressed. All these parameters are investigated properly in our study, appreciably and reliably.

### 3.7. Analyzing of Human Samples

To use the fabricated sensor probe for its real purpose, it was employed to analyze human samples (blood serum & urine) by the recovery method. The recovery method is way to test the applicability of a newly assembled nanomaterial sensor probe, where a sample with known concentration is analyzed. The resultant value is compared with the known value, in terms of percentage of recovery. In this study, real samples of known concentrations were prepared. The human fluid (urine and blood serum) was added and then subjected to investigation with the fabricated sensor probe. The investigation results, by applying DPV and CV as presented in Table 2, were found satisfactory and acceptable. From these observation, it is introduced a new route for the determination of unsafe biochemicals by using various nanoscale or nanocomposite materials fabricated novel sensor probe onto glassy carbon electrode by electrochemical approach [55,56,57,58] for the safety of healthcare as well as biomedical fields in a broad scale.

## 4. Conclusions

In this approach, the UA sensor probe was assembled with wet-chemically prepared PbO-NiO NCs coated onto GCE. The prepared PbO-NiO NCs sensor-probe was used for the detection of UA in the pH = 7.0 buffer by using DPV and CV electrochemical methods. The obtained sensor parameters including LDR, sensitivity, LOD, reproducibility, response time, and stability were investigated and found to return good and satisfactory results. In addition, the UA sensor-probe successfully analyzed human samples by the recovery method. Thus, it introduces an excellent method for the detection of human bio-chemicals applying doped microstructure materials as sensing substrates, with practical potential in the healthcare sector and biomedical fields on a broad scale.

## Figures and Tables

**Table 1:** Comparison of UA sensors based on various nanomaterial/nanocomposite materials by electrochemical approach. This study is bold.

Electrode Materials	Sensor Type	Detection Method	* DOL	*^#^* LDR	Sensitivity	Refs.
-	Non-Enzymatic	HPLC-Amperometry	5.1 µM	0.06~6 µM	-	[12]
-	Non-Enzymatic	fluorometric	2.86 μM	0.01~1 mM	-	[13]
-	Enzymatic	fluorometric	-	0.03~0.6 mM	-	[14]
Nafion/CPE	Non-Enzymatic	Voltammetric	0.25 μM	0~50 μM	-	[26]
PGE	Non-Enzymatic	Voltammetric	1.4 µM	-	7.7 µAµM^−1^cm^−2^	[27]
EDOT/AuE	Non-Enzymatic	DPV	1.5 μM	2.0~600 μM	4.05 AM^−1^cm^−2^	[30]
PZ–Silica matrix	Non-Enzymatic	Voltammetric	3.7 μM	22~350 μM	-	[31]
PSR/CPE	Non-Enzymatic	cyclic voltammetric	0.176 µM	0.3~3.1 µM	-	[48]
erGO/ITO	Non-Enzymatic	DPV	0.3 μM	0.3~100 μM	-	[49]
N-doped GO/GCE	Non-Enzymatic	DPV	0.045 nM	0.1nM~0.02 µM	-	[50]
Uricase/ZnO/GCE	Enzymatic	DPV	2.0 µM	5.0 µM~0.1 mM	-	[51]
ZnNi@f-MWCNT/GCE	Non-Enzymatic	DPV	-	0.2–1.1 mM	-	[52]
N-rGO/GCE	Non-Enzymatic	DPV	0.2 µM	1~30 µM	-	[53]
Au/RGO/GCE	Non-Enzymatic	DPV	1.8 µM	8.8~53.0 µM	-	[54]
PbO/NiO NCs/GCE	**Non-Enzymatic**	**DPV**	**43.0 µM**	**0.15** **~** **1.35 mM**	**0.22 µAµM^−1^cm^−2^**	**This**
	**Non-Enzymatic**	**CV**	**41.0 µM**	**0.15** **~** **1.35 mM**	**0.23 µAµM^−1^cm^−2^**	**study**

* DL (detection limit), ^#^ LDR (linear dynamic range), µM (micromole), mM (millimole).

**Table 2:** Analyses of real samples with PbO-doped NiO NCs/GCE probe sensor.

Methods	Real Samples	Added UA Conc. (mM)	Measured UA Conc. ^a^ by PbO-NiO NCs/GCE (mM)			Average Recovery ^b^ (%)	RSD ^c^ (%) (n = 3)
			R1	R2	R3		
DPV	Blood serum	0.5000	0.4956	0.4916	0.4909	98.54	2.07
	Urine	0.5000	0.4937	0.4907	0.4987	98.81	2.15
CV	Blood serum	0.5000	0.5016	0.4973	0.5108	100.64	1.52
	Urine	0.5000	0.4892	0.4908	0.4934	98.23	1.73

^a^ Mean of three repeated determination (signal to noise ratio 3) with PbO-NiO NCs/GCE. ^b^ Concentration of UA determined/concentration taken (Unit: µM). ^c^ Relative standard deviation value indicates precision among three repeated measurements (R1, R2, & R3).

## Data Availability

Data are contained within the article.
